# The Buried Bumper Syndrome: A Catastrophic Complication of Percutaneous Endoscopic Gastrostomy

**DOI:** 10.7759/cureus.4330

**Published:** 2019-03-27

**Authors:** Jinendra Satiya, Akiva Marcus

**Affiliations:** 1 Internal Medicine, University of Miami, John F Kennedy Regional Campus, Atlantis, USA; 2 Gastroenterology, University of Miami, John F Kennedy Regional Campus, Atlantis, USA

**Keywords:** peg, complications of peg, buried bumper syndrome, enteral tube feeding

## Abstract

Percutaneous endoscopic gastrostomy (PEG) is a safe and widely used method of providing enteral nutrition in patients unable to tolerate per oral intake. Common complications include gastrointestinal bleeding, dislodgment, perforation, abdominal wall abscess, and aspiration. “Buried bumper syndrome” (BBS) is a rare but potentially fatal complication resulting in malfunction of the tube, gastric perforation, bleeding, and peritonitis. Gastroenterologists should be cognizant of the clinical presentation and treatment of a buried bumper. We report a case of a 56-year-old woman who presented with coffee-ground emesis and was managed with the placement of a gastro-jejunal tube.

## Introduction

Gauderer and Ponsky first discovered percutaneous endoscopic gastrostomy (PEG) as a means of providing nutrition delivery in patients with oropharyngeal dysphagia [1]. The first case of "buried bumper syndrome" (BBS) was reported by Nelson and treated with endoscopic forceps [2]. Prompt recognition of the syndrome is essential in providing the appropriate treatment to prevent clinical deterioration. The diagnosis should be suspected when a patient with a PEG tube presents with pain and erythema and swelling around the PEG tube insertion site. These patients should be admitted to the hospital and resuscitated with intravenous fluids and antibiotics. Removal of the tube is the ultimate treatment and can be achieved by either endoscopy or surgery.

## Case presentation

A 56-year-old female with a history of primary biliary cirrhosis, disease-related muscular weakness, and progressive respiratory failure with a tracheostomy presented from a nursing home with coffee ground emesis and respiratory distress. A percutaneous endoscopic gastrostomy tube was placed six months ago without any complications. On examination, the abdomen was soft, mildly tender to palpation around the PEG insertion site without any signs of peritonitis. Leakage around the PEG tube was noted with surrounding erythema and swelling. Granulation tissue was visible arising from the PEG tube tract. Laboratory examination showed 10,500 white blood cells/µL (70% neutrophils) and a hemoglobin of 12 gms/dl.

**Figure 1:**
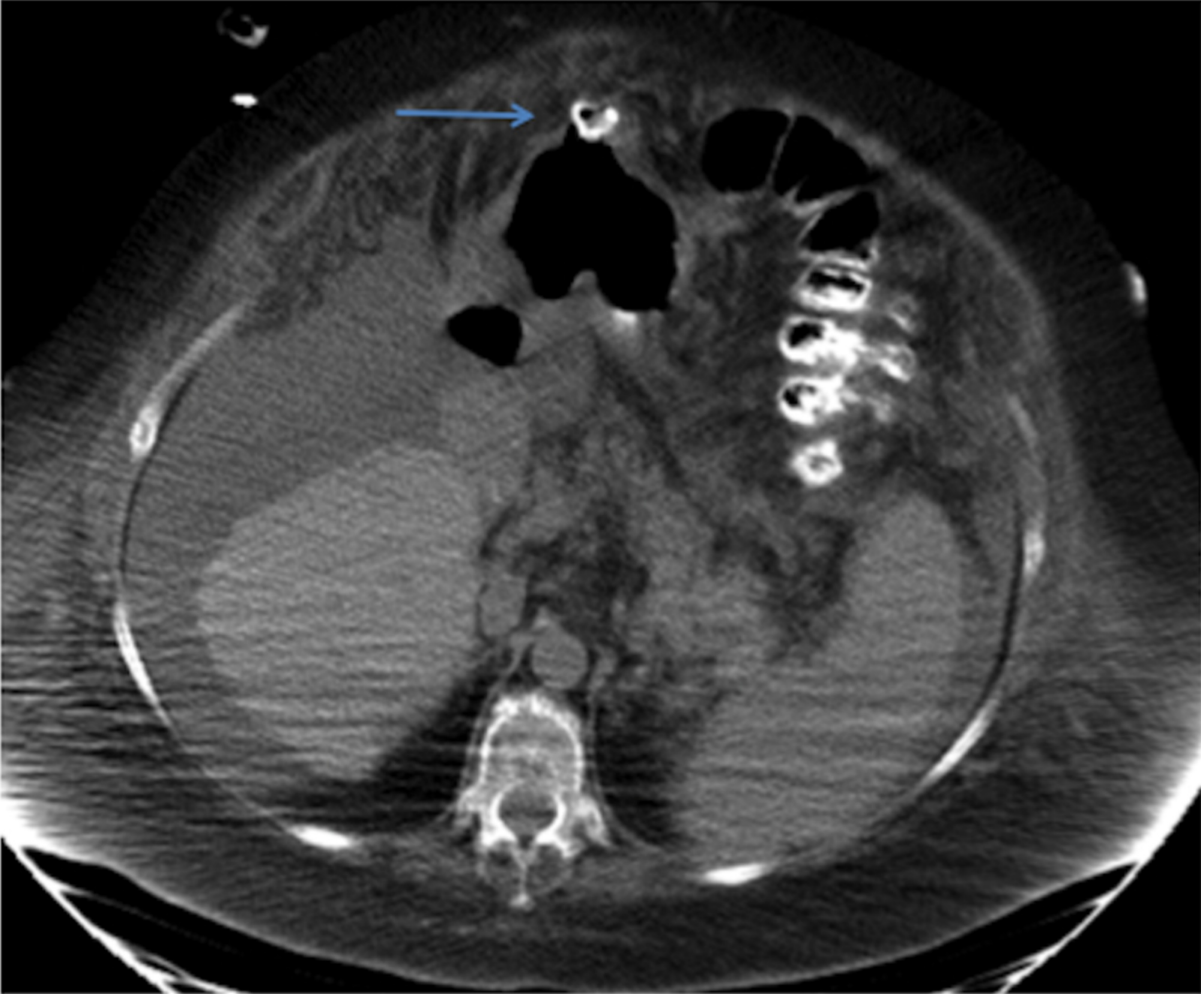
An axial view of the computed tomography scan of the abdomen demonstrating the erosion of the percutaneous endoscopic gastrostomy (PEG) tube through the stomach wall i.e. "buried bumper"

**Figure 2:**
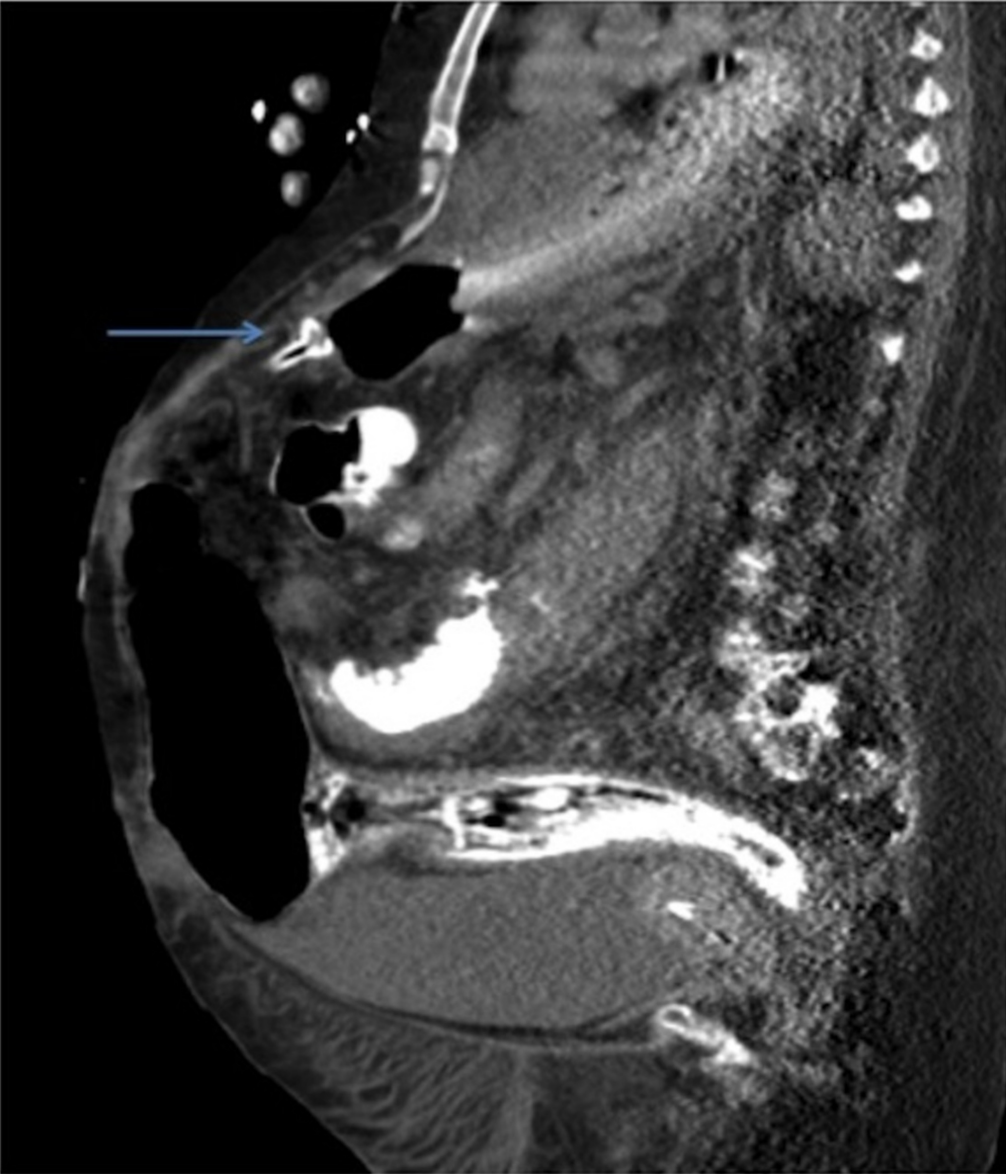
The erosion of the gastrostomy tube through the stomach wall depicted on coronal view of a computed tomography (CT) scan of the abdomen

**Figure 3:**
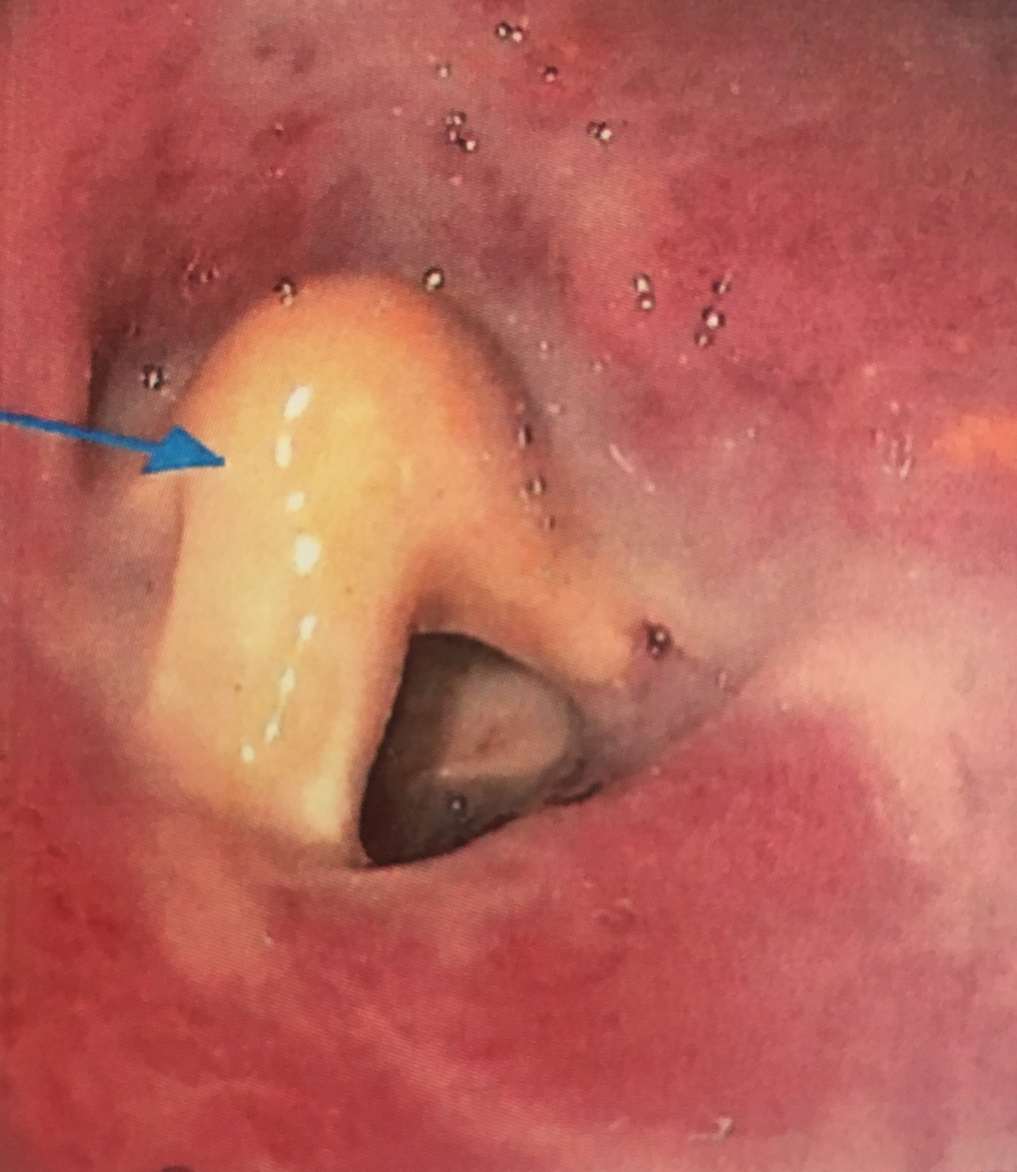
Erosion of the gastrostomy tube through the stomach wall visualized on upper endoscopy

A computed tomography (CT) scan of the abdomen demonstrated erosion of the gastrostomy tube through the stomach wall (Figures 1-2). A contrast study was not performed given the CT scan findings, for fear of causing peritonitis. An upper endoscopy revealed a gastrostomy tube whose internal bumper was found to be eroding into the wall of the gastric antrum (Figure 3). There was surrounding erythema, inflammation, and ulceration. No signs of free perforation were seen on endoscopy. The patient was managed with removal of the existing gastrostomy tube and placement of a gastro-jejunal tube at another site, both performed by interventional radiology. The patient reported significant pain relief after the procedure and was discharged to complete a five day course of ciprofloxacin.

## Discussion

Percutaneous endoscopic gastrostomy (PEG) was introduced in 1980 by Gauderer et al. and provides a well-tolerated option for long-term enteral feeding [1]. Indications for a PEG tube include neurological diseases such as Parkinson's disease, cerebral palsy, multiple sclerosis, cerebrovascular disease, head injury, head and neck cancers, congenital anomalies such as trachea esophageal fistula, cystic fibrosis, facial surgery, burns, short bowel syndrome, and chronic renal failure. Contraindications to the placement of a PEG tube include coagulopathies, hemodynamic instability, sepsis, massive ascites, peritoneal carcinomatosis, and refractory gastroparesis. A PEG tube should be removed as soon as it is not needed or when complications such as "buried bumper syndrome" (BBS) occur [3].

Complications of PEG placement can be classified as early or late. Early complications include those related to the procedure such as bleeding, aspiration during the procedure, and bowel perforation. Late complications include BBS, persistent leakage around the PEG site, and the development of gastrocolocutaneous fistulas. Most cases of BBS occur after one year, although earlier presentations may also be encountered [4].

"Buried bumper syndrome" was first described in 1988. Risk factors of BBS can be categorized as relating to the patient (comorbidities, manipulation of PEG tube, and medications); procedure (position of external fixator, excess tension at the external fixator site, dressing, and location); or care of the PEG tube (changing of the position of the external fixator). Incidence of BBS is reported at 0.3%-2.4% [5]. Symptoms include pain around the insertion site, leakage, erythema, and swelling around the PEG tube indicating infection. Patients can also present with severe sepsis, gastrointestinal bleeding or peritonitis [6]. It is important to note that patients might be asymptomatic early in the pathological course even when the tube is non-functional. Occasionally, the migrated internal bumper might be palpable on abdominal examination [7]. Abdominal wall abscesses have also been reported with one death secondary to BBS [8]. Physical examination can demonstrate non-rotation of the PEG tube or inability to slide through the stoma, which is key in clinching a diagnosis of BBS and helping to distinguish it from other PEG tube complications [9].

The main etiology is thought to be excessive tension between the internal and external bumpers leading to subsequent mucosal ischemia and necrosis [10]. The diagnosis of BBS is a clinical one, with imaging studies helpful in identifying the exact location of the bumper. As soon as the diagnosis is suspected, the PEG tube should not be used further to administer fluids, medications, or feeds. The patient should be admitted to the hospital and resuscitated with intravenous fluids. Antibiotics are recommended even in the absence of infection given the fact that the pathophysiology of the disease process presumes contamination of the abdominal wall with tube feeds [6]. An upper endoscopy is indicated for all suspected cases of BBS. It can reveal a pressure ulcer below the disc and mucosa overgrowing the disc. The surrounding mucosa may be edematous. The modality of treatment is determined by the depth of disc migration from the lamina muscularis propria of the stomach, which can be estimated by ultrasonography, computed tomography, or endoscopic ultrasound [5].

The average life span of a PEG tube is one to two years. Various methods have been described to replace the PEG tube in the case of BBS. Two classification systems were introduced in 2015 to help guide the management of BBS. Cyrany et al. graded BBS into five stages based on depth estimation by abdominal ultrasound [5]. The first four stages were amenable to endoscopic treatment with stage five requiring surgical intervention. Richter-Schrag et al. recommended endoscopic therapy for stages II-IV [11]. ​​​​ For most gastrostomy tubes that have soft internal retention devices, simple extraction can be performed. When required, a new PEG tube can be placed simultaneously at the time of removal of the old one. Placing a new PEG tube and removing the old one by simple internal extraction is the treatment of choice in cases of incomplete BBS (partially covered). A bougie can also be used to remove a partially covered buried bumper with attempts made to push it back into the stomach. If this is unsuccessful, the papillotome technique described by Mueller-Gerbes et al. can be safely employed for removal [12].

For complete BBS, cutting with a needle-knife is the procedure of choice. It is important to remember that the gastrocutaneous tract formed by a PEG tube takes about four to six weeks to mature after placement. Hence, a PEG tube dislodged within 30 days from placement should always be replaced by endoscopy [13]. In the rare event of an intraperitoneal tube placement, exploratory laparotomy is required as these can cause peritonitis and be potentially fatal. Kejariwal et al. recommended a conservative approach entailing the “cut and leave it” strategy, in patients that are deemed poor surgical candidates and have a very poor prognosis [14].

Endoscopic therapy includes the ''push-pull T-technique'' employed by Boyd et al., argon plasma coagulation for destruction of granulation tissue, and the innovative techniques of "natural orifice transluminal endoscopic surgery" (NOTES) [15,16,17]. The HookKnife is a recent technique developed by Wolpert et al. [18]. Crowley et al. described a radiological technique entailing the use of an angioplasty catheter under fluoroscopic control [19]. Laparoscopy can be performed for discs that are buried inside the stomach [20]. Preventive measures include ensuring a 3-5 mm free space between the skin level and the external bumper. Close follow-up with a nutrition team is key.

## Conclusions

A rare complication of PEG placement is buried bumper syndrome, which occurs when the internal bumper migrates through the gastric wall. Despite a low prevalence, BBS can lead to peritonitis and be potentially fatal. Difficulties in feeding through the PEG tube, leakage around the tube, and patient discomfort are symptoms that should alert the clinician to a possibility of BBS. Treatment options range from simple endoscopic replacement, placement of a gastro-jejunal tube, as was done in the case of our patient, to surgical laparotomy.
